# Comparison of Women from Georgia and Contiguous States Who Obtained Abortions in Georgia, 1994–2016

**DOI:** 10.1007/s10995-019-02863-9

**Published:** 2020-01-16

**Authors:** Rachel Shapiro, Blake Erhardt-Ohren, Roger Rochat

**Affiliations:** Emory University Rollins School of Public Health, Atlanta, GA USA

**Keywords:** Abortion, US southeast, Medical travel, Reproductive health

## Abstract

**Objectives:**

To determine trends for Georgia and contiguous state residents seeking abortions in Georgia between 1994 and 2016.

**Methods:**

We analyzed aggregate vital statistics data, collected in Georgia, on Georgia residents (*n* = 675,995) and contiguous state residents (Alabama, Florida, North Carolina, South Carolina, Tennessee) (*n* = 76,232) obtaining abortion and delivery services in Georgia between 1994 and 2016. We examined demographic, pregnancy, and abortion characteristics using counts, ratios, and *χ*^2^ tests of proportion.

**Results:**

Of the data analyzed, 10.1% of all abortions were for contiguous state residents. The number of abortions in Georgia for contiguous state residents increased 35.3% from 1994 to 2016 (from *n* = 3115 to *n* = 4216) while it decreased for Georgia residents by 11.1% (from *n* = 32,934 to *n* = 29,264). Contiguous state residents exhibited a higher abortion ratio (1115) compared to Georgia women (224). These populations exhibited statistically significant differences across all variables and time points. Both populations demonstrated similar trends in ethnicity, race, education, marital status, and age. However, contiguous state residents were more likely to obtain an abortion at ≥ 20 weeks gestational age (13.8%) and obtained a lower proportion of suction curettage abortions (60.0%) and a higher proportion of dilation and evacuation procedures (31.9%). They were also less likely to be primigravid.

**Conclusions for Practice:**

Women from neighboring states seek abortions in Georgia later in gestation and may therefore lack affordable, safe, early abortion care in their home states. Understanding trends in travel for abortion can allow providers and policymakers to better respond to the needs of patients.

## Significance

Due to the current policy environment, including the passage of a 2019 “heartbeat” bill in Georgia, evaluating trends associated with abortion care provides timely and important information on women who access Georgia’s abortion services. This article examines trends in vital statistics data on abortion for Georgia women obtaining abortions in Georgia (*n* = 675,995) compared to women from contiguous states traveling to receive abortions in Georgia (*n* = 76,232). The findings suggest that contiguous state women preferentially sought abortion services in Georgia compared to delivery services and differed from Georgia residents in gestational age, abortion procedure, and gravidity.

## Introduction

Patients seek a variety of health care services outside of their state of residency, whether due to proximity, quality of care, service availability, anonymity, or related factors—abortion is one of these services (Jatlaoui et al. 2018). Women seeking abortions face a number of difficulties that may contribute to their decision to travel for services. Because abortion laws vary among states, access depends in part on a woman’s location. Between 2011 and 2017, 32 states passed 394 restrictions on abortion (Nash and Dreweke 2019). As of September 2019, 43 states had gestational age limits and 27 states specified a mandatory waiting period (An Overview of Abortion Laws 2019). In the first quarter of 2019, 28 states introduced abortion ban legislation, seven of these bills passed at least one state legislative chamber, and in three states they became law (Nash et al. 2019). These bills included trigger bans to outlaw abortion if *Roe v. Wade* is overturned, gestational age bans, reason bans (based on fetal characteristics), and bans on certain abortion procedures (Ibid 2019). Such differences in policies may lead to unequal access to abortion services based on a woman’s state of residency.

In the southern United States, abortion access remains especially restricted. Between 2011 and 2017, the South and Midwest accounted for 86% of all abortion restrictions nationwide (Nash and Dreweke 2019). During this period, 180 abortion restrictions were enacted, and 50 clinics were closed in the South (Ibid 2019). An analysis of abortion policy in 2018 found that of 27 states classified as having a “hostile” policy environment for abortion provision, 13 were located in the South (Jones et al. 2018). Abortion policies in the South continue to rapidly change. Of the 28 states introducing abortion ban legislation in the first quarter of 2019, 10 were southern (Nash et al. 2019). In May 2019, both Mississippi and Georgia passed laws that established a detectable fetal heartbeat as the gestational age limit for abortions (H.R. 732, 2019; H.R. 481, 2019). Also in May 2019, Alabama passed an abortion ban, with no exceptions for rape and incest (H.R. 314, 2019).

Many women across the South also face geographical barriers to abortion care. More than 43.3% of women seeking abortion in the South travel more than 25 miles for services, with 9.8% traveling more than 100 miles (Jones and Jerman 2017). The median distance from a woman’s census block group to the nearest clinic ranged from 6.2 miles in Maryland to 68.8 miles in Mississippi (Bearak et al. 2017). The majority of “abortion deserts”—cities where women must travel further than 100 miles for services—are located in the South or Midwest (Cartwright et al. 2018). Lack of proximity to services often results in additional challenges to access, including elevated costs and delays in obtaining care (Barr-Walker et al. 2019 and Jerman et al. 2017).

Due to limited clinic availability, restrictive abortion policies, and other factors, women travel between states to seek abortion services (Centers for Disease Control 2015). However, few scholars have focused on whether patients crossing state lines for abortion differ from those obtaining abortions in their state of residency. We seek to fill this gap by examining demographic and abortion trends across time for women traveling for abortion in the southeastern United States. We compared trends and characteristics of Georgia residents obtaining abortions in Georgia with contiguous state residents (Alabama, Florida, North Carolina, South Carolina, and Tennessee) travelling to Georgia for abortion services between 1994 and 2016. To determine whether abortion-related travel followed similar patterns as travel for other obstetric services, we analyzed trends in travel to Georgia for delivery services compared to abortion services. Our objective was to understand the characteristics of populations obtaining abortions and to identify trends in abortion services over time. Given that comparatively little is known about women traveling for abortion services, this study provides a baseline for evaluating legal and policy changes and information to help policymakers and providers support patients’ ability to obtain high-quality abortion care.

## Methods

### Data Source and Definitions

We examined surveillance data in Induced Termination of Pregnancy (ITOP) and Birth files from the Georgia Department of Public Health (GDPH) between 1994 and 2016; all data included in this study refer to events that occurred in Georgia. Data for all periods and variables were aggregated to state-level (Alabama, Florida, Georgia, North Carolina, South Carolina, and Tennessee). This analysis did not use individual patient data. Variables included age (< 20, 20–29, 30–39, or 40–55 years), marital status (married or unmarried), education completed (less than 9th, 9–11th, high school graduate or GED completed, or some college or higher), race (white, black, or other), and ethnicity (Hispanic or non-Hispanic). For abortion characteristics, we included gestational age from last menstrual period (LMP) (< 20 weeks, ≥ 20 weeks), abortion method (suction curettage, sharp curettage, dilation and evacuation [D&E], mifepristone, or other), abortion service location (Atlanta or non-Atlanta), and first pregnancy (yes or no). Throughout the paper, we use the original terminology from the GDPH dataset, with the exception of ITOP, which we refer to as abortion.

### Analysis

We first calculated counts and percentages for each variable. We examined eight variables for all six states involved in the analysis in addition to pooled data for residents of contiguous states. We analyzed annual changes across the study period by comparing contiguous state resident data with Georgia resident data to identify trends. We then calculated the abortion ratio (the number of abortions obtained per 1000 live births) for both populations to compare trends for delivery services compared to abortion services. Finally, we used SAS® 9.4 software to calculate the *χ*^2^ test of proportions to determine if, for each year and variable, Georgia and contiguous states residents were statistically significantly different, defined at a *p* value of < 0.05.

### Missing and Excluded Data

Changes in the reporting system led to an increase in unknown data for certain variables over the 23-year period. Unknown data for each variable comprised between 0 and 38% of all data collected for certain variables for residents of each state under analysis, with education and ethnicity variables having the highest percentage of unknown data. These data were excluded in the analysis due to the uniformity of the drop across all affected years for each variable.

## Results

### Abortion Count Trends

**Fig. 1:**
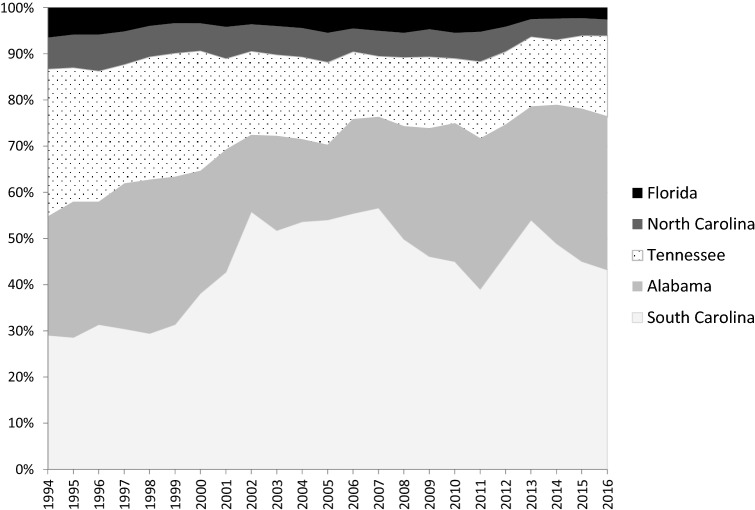
Percent distribution of contiguous state residents’ abortions in Georgia, by State, 1994–2016

We analyzed 752,227 abortions in Georgia between 1994 and 2016, 675,995 (89.9%) for Georgia residents and 76,232 (10.1%) for contiguous state residents. 35.3% more women from contiguous states received abortion services in Georgia in 2016 than in 1994 (3115 to 4216), although trends in individual states varied. Nearly three-fourths (70.5%) of contiguous state residents coming to Georgia for abortion came from Alabama (*n* = 19,689) and South Carolina (*n* = 34,027). Residents from North Carolina and Tennessee obtained 4409 and 14,834 abortions, respectively, in Georgia. Florida residents comprised the smallest contributor to abortions in Georgia, with only 3273 abortions over the 23-year period. Figure 1 displays the proportion of abortions for each contiguous state in Georgia.

Alabama and South Carolina residents’ reliance on Georgia for abortion services increased throughout the study period 74.8% (800 to 1398) and 99.0% (905 to 1801), respectively. Florida, North Carolina, and Tennessee residents obtaining abortions in Georgia decreased during the study period: − 44.0% (200 to 112), − 26.9% (212 to 155), and − 24.8% (998 to 750). A higher proportion of residents from all contiguous states except South Carolina sought abortions in the Atlanta region compared to elsewhere in Georgia. Women from Florida, North Carolina, and Tennessee traveled to Atlanta more than 90% of the time (91.7%, 95.7%, and 97.9%, respectively). Alabama women obtaining services went to Atlanta 71.8% of the time and South Carolina residents travelled to Atlanta 22.4% of the time.

**Fig. 2:**
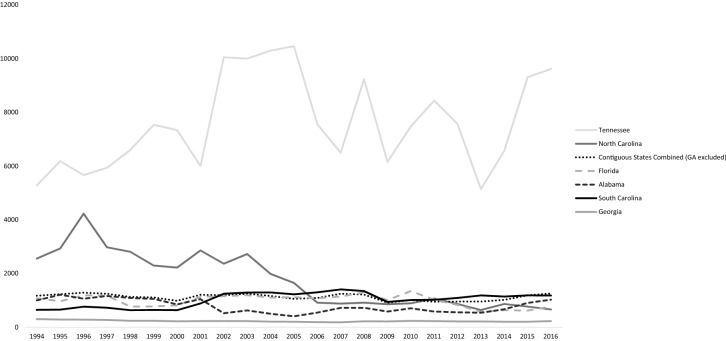
Abortion to live birth ratio (ALB) for Georgia residents, residents of individual contiguous states, and contiguous state residents combined, 1994–2016

Residents of Georgia averaged an abortion ratio of 224 over the study period, compared to a ratio of 1115 for contiguous state residents. Tennessee had the highest abortion ratio of 7159 (range 5140 to 10,464), and North Carolina had the second highest abortion ratio ranging between 4228 in 1996 and low of 641 in 2013. Alabama, Florida, and South Carolina abortion ratios remained within the range of 1405 (South Carolina, 2007) and 404 (Alabama, 2005). The abortion ratios of all states included in the study, over time, can be found in Fig. 2. These values reveal that women in contiguous states travel to Georgia more often for abortions than for delivery services.

### Demographic Variables

Demographic data are displayed in Tables 1 and 2*,* and are categorized by state of residency and time period. When examining contiguous states separately, all exhibited similar trends in ethnicity, education, marital status, and maternal age across time. The proportion of black women receiving abortions increased for Georgia residents from 52.7% (17,351) in 1994 to 68.9% (18,110) in 2016 and for contiguous state women from 37.0% (1152) in 1994 to 60.4% (2400) in 2016. The proportion of Hispanic patients who received abortion services between 1994 and 2016 increased in each state, from 2.6 to 7.4% among Georgia residents and 0.9% to 4.5% among contiguous state residents.

**Table 1:** Demographic and abortion characteristics for Georgia women receiving abortions in the State of Georgia, 1994–2016

	1994–1999		2000–2005		2006–2011		2012–2016	
Total number of abortions—*n*	187,172		175,228		176,467		137,128	
Total number of live births—*n*	704,245		814,334		852,910		650,672	
Abortion to live birth ratio^a^	265.8		215.2		206.9		210.7	

	Count—*n*	Valid percent	Count—*n*	Valid percent	Count—*n*	Valid percent	Count—*n*	Valid percent
*Age group*								
19 and under	34,917	18.7	27,082	15.5	24,525	13.9	13,619	9.9
20–29	106,806	57.1	99,886	57.0	99,962	56.6	79,543	58.0
30–39	41,365	22.1	43,526	24.8	46,507	26.4	39,000	28.4
40–55	4084	2.2	4734	2.7	5473	3.1	4966	3.6
*Race*								
Black or African-American	101,186	54.1	98,484	56.2	103,163	64.1	82,173	67.1
White	80,806	43.2	68,413	39.0	48,938	30.4	32,175	26.3
Other	5180	2.8	8331	4.8	8823	5.5	8071	6.6
*Ethnicity*								
Not Hispanic	175,996	97.4	152,684	93.7	143,379	87.5	118,355	92.6
Hispanic	4687	2.6	10,319	6.3	20,411	12.5	9516	7.4
*Marital status*								
Married	34,067	18.4	32,098	18.7	28,286	18.4	19,759	15.8
Unmarried	151,450	81.6	139,313	81.3	125,038	81.6	105,542	84.2
*Education*								
Less than 9th	3615	2.0	5139	3.0	3027	1.9	4141	3.3
9th thru 12th, no diploma	23,300	12.8	19,413	11.5	18,319	11.8	13,106	10.5
High school graduate or GED completed	74,828	41.0	69,708	41.4	58,649	37.8	39,420	31.7
Some college or higher	80,719	44.2	74,317	44.1	75,290	48.5	67,832	54.5
*Gestational age*								
Under 20 weeks	183,727	98.2	171,738	98.0	171,281	97.8	134,263	98.0
20 weeks and over	3445	1.8	3490	2.0	3941	2.2	2764	2.0
*First pregnancy*								
No	133,471	71.5	127,908	73.3	122,940	73.2	93,498	71.2
Yes	53,155	28.5	46,512	26.7	45,019	26.8	37,843	28.8
*Pregnancy termination method*								
Suction curettage	170,525	91.1	157,423	91.1	146,115	84.4	86,814	63.3
Sharp curettage	73	0.0	76	0.0	666	0.4	411	0.3
Dilation and evacuation	15,847	8.5	14,142	8.2	15,483	8.9	19,974	14.6
Other	727	0.4	1150	0.7	1877	1.1	1696	1.2
Mifepristone	0	0.0	0	0.0	8956	5.2	28,171	20.6

These categories were not used for data analysis, but rather serve to condense the table for insertion in the manuscript. All percent measures are "valid percents"—this means that unknown values are excluded from the denominator

^a^Abortion to live birth ratio is calculated by total number of abortions divided by total births multiplied by 1000

**Table 2:** Demographic and abortion characteristics for women from states contiguous to Georgia receiving abortions in the state of Georgia, 1994–2016

	1994–1999		2000–2005		2006–2011		2012–2016	
Total number of abortions—*n*	18,142		20,150		19,767		18,173	
Total number of live births—*n*	15,222		17,645		18,579		16,935	
Abortion to live birth ratio^a^	1191.8		1142.0		1063.9		1073.1	

	Count—*n*	Valid percent	Count—*n*	Valid percent	Count—*n*	Valid percent	Count—*n*	Valid percent
*Age group*								
19 and under	5085	28.0	4243	21.1	3827	19.4	2198	12.1
20–29	9845	54.3	11,542	57.3	11,300	57.2	10,569	58.2
30–39	2890	15.9	3896	19.3	4162	21.1	4834	26.6
40–55	322	1.8	469	2.3	478	2.4	572	3.1
*Race*								
Black or African-American	7384	40.7	9824	48.8	10,028	54.3	9743	57.9
White	10,439	57.5	9871	49.0	7831	42.4	6500	38.7
Other	319	1.8	455	2.3	597	3.2	570	3.4
*Ethnicity*								
Not Hispanic	17,740	99.1	18,528	96.9	17,381	93.1	16,504	95.5
Hispanic	158	0.9	589	3.1	1,281	6.9	781	4.5
*Marital status*								
Married	2676	14.9	3177	16.1	2964	16.3	2504	14.6
Unmarried	15,281	85.1	16,575	83.9	15,240	83.7	14,619	85.4
*Education group*								
Less than 9th	481	2.7	582	3.0	457	2.6	462	2.7
9th thru 12th, no diploma	3045	17.0	2787	14.2	2645	14.8	1811	10.6
High school graduate or GED completed	7222	40.4	7472	37.9	6167	34.5	5564	32.5
Some college or higher	7140	39.9	8849	44.9	8590	48.1	9286	54.2
*Gestational age group*								
Under 20 weeks	15,255	84.1	17,385	86.3	16,639	84.7	16,353	90.0
20 weeks and over	2887	15.9	2765	13.7	3016	15.3	1811	10.0
*First pregnancy*								
No	11,279	62.4	13,797	68.9	13,206	69.2	12,577	70.5
Yes	6799	37.6	6239	31.1	5889	30.8	5255	29.5
*Pregnancy termination method*								
Suction curettage	11,035	61.7	14,121	72.5	12,892	67.5	6767	37.3
Sharp curettage	11	0.1	5	0.0	89	0.5	115	0.6
Dilation and evacuation	6826	38.2	5224	26.8	5003	26.2	6748	37.1
Other	4	0.0	123	0.6	249	1.3	102	0.6
Mifepristone	0	0.0	0	0.0	870	4.6	4434	24.4

These categories were not used for data analysis, but rather serve to condense the table for insertion in the manuscript. All percent measures are "valid percents"—this means that unknown values are excluded from the denominator

^a^Abortion to live birth ratio is calculated by total number of abortions divided by total births multiplied by 1000

Education and marital status were similar among Georgia and contiguous state residents, while the proportion of first pregnancy differed between the two populations. The proportion of college-educated women increased while the proportion of women with 9–11th grade education decreased. For both Georgia residents and contiguous state residents, abortion trends by marital status remained stable between 1994 and 2016; married women comprised 13.8–19.4% of all Georgia residents and 12.8–17.6% of all contiguous state residents obtaining abortions in Georgia annually. Both populations had similar trends for maternal age, including a decrease in the proportion of women < 20 years of age, from 18.8 to 8.6% for Georgia residents and from 28.9 to 10.4% for contiguous state residents. The proportion of primigravid women decreased steadily throughout the 23-year study period in all contiguous states, from 40.4 to 28.9%, while it remained stable (25.2–29.8%) for Georgia residents during the study period.

**Fig. 3:**
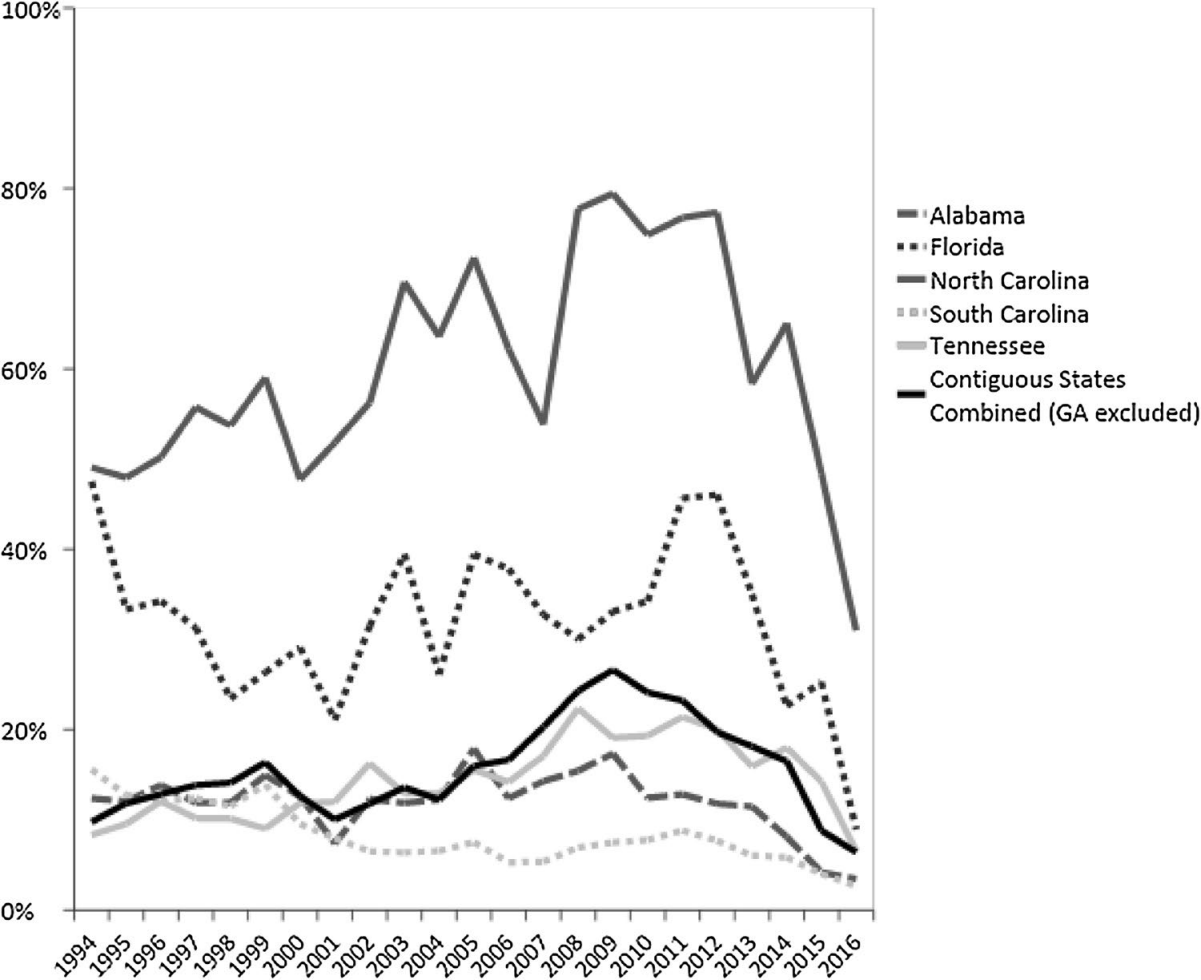
Proportion of abortions at a gestational age of ≥ 20 weeks for contiguous state residents, combined and individually, 1994–2016

Across all time points, between 97.5 and 98.6% of abortion procedures took place at a gestational age of < 20 weeks for Georgia residents; this proportion ranged from 82.1 to 95.2% for contiguous state residents receiving abortions in Georgia. Only 2.0% of procedures were at ≥ 20 weeks gestational age for Georgia residents, compared with 13.8% for women from contiguous states. Figure 3 displays the percentage of abortions that were at a gestational age of ≥ 20 weeks for contiguous state residents.

**Fig. 4:**
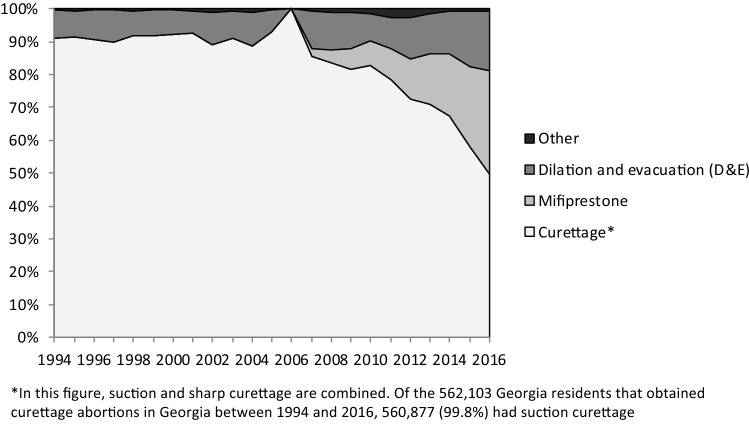
Trends in abortion methods for Georgia residents in Georgia, 1994–2016

**Fig. 5:**
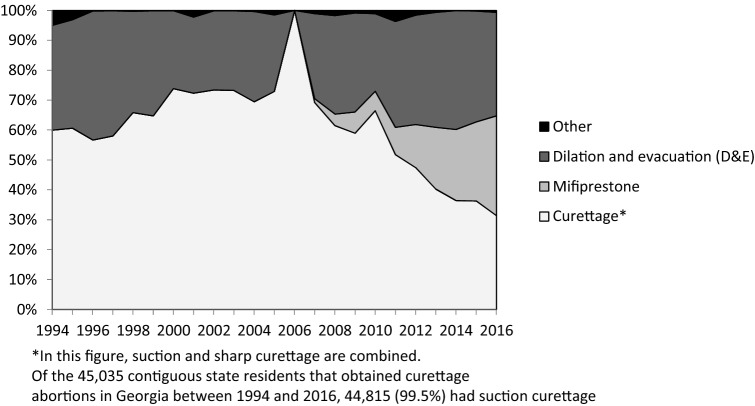
Trends in abortion methods for contiguous state residents in Georgia, 1994–2016

Across the 23-year period, suction curettage was the most common abortion procedure, accounting for 83.7% of all procedures for Georgia residents and 60.0% for contiguous state residents. All states experienced a decrease in the use of suction curettage over time but had upticks in its use in 2006 and 2010–2011. By 2016, mifepristone had become the second-most common abortion procedure for residents of all states in the study. In 2016, the highest proportion of mifepristone use was among South Carolina residents (38.8%), and the lowest proportion was among North Carolina residents (16.1%). D&E procedures remained consistent, until 2007, when they began to increase. Figures 4 and 5 display the relative proportions of different abortion procedures for Georgia and contiguous state residents.

While Georgia and contiguous state residents had similar trends for mifepristone, curettage, and D&E, the proportional use of these methods differed at various time points. Georgia residents had the highest proportion of suction curettage in 1994 at 91.2%, which decreased to 49.8% by 2016. North Carolina residents had the lowest use of suction curettage in 1994 at 14.6%, which increased to 27.1% by 2016. D&E procedures were utilized in a lower proportion by residents from Georgia (9.8%) compared to women from contiguous states (31.9%).

### Results of Statistical Testing

In all time periods, our populations were statistically significantly different for all variables, except the following instances in which the populations were not statistically significantly different for one variable in one year: marital status in 2006, 2007, 2013, 2014, and 2015; first pregnancy in 2013–2015; and education completed in 2012, 2013, and 2015.

## Discussion

Our objective was to examine demographic and abortion trends across time for women obtaining abortions in Georgia, including those travelling for services. Across the time period under review, Georgia was an important source of abortion services, especially for non-residents. CDC abortion surveillance data demonstrates that women across the United States travel between states to obtain abortion services (Jatlaoui et al. 2018). Of the data analyzed, 10.1% of abortions between 1994 and 2016 in Georgia were for residents of contiguous states, 70.5% of which were for residents from South Carolina and Alabama.

Contiguous state residents obtained abortion services in Georgia more frequently than delivery services. Compared to a national abortion ratio of 186 abortions per 1000 live births (Cartwright et al. 2018), contiguous state residents obtaining abortions in Georgia had an abortion ratio of 1115 per 1000 live births (compared to 224 for Georgia residents). National abortion trends demonstrate a decrease in the total number of abortions in the United States (Jones and Jerman 2017). Hypotheses for this decline include a decrease in pregnancies and the rise of self-managed abortions (Nash and Dreweke 2019). While our data demonstrate an 11.1% decrease in abortions for Georgia residents (from 32,934 to 29,264), they show that the number of contiguous state residents obtaining abortion services in Georgia increased 35.3% from 1994 to 2016 (3115 to 4216). Trends for individual states varied. The number of abortions obtained by women from South Carolina increased, while it remained stable for North Carolina and Florida women. In comparison, the number of abortions for Alabama and Tennessee residents initially declined until 2005 (Alabama) and 2009 (Tennessee) before increasing in subsequent years.

Both Georgia and contiguous state residents obtaining abortions in Georgia exhibited similar demographic changes across time. The exception was gravidity; while the proportion of women aborting a first pregnancy decreased across time for contiguous state residents, it remained stable for Georgia residents. For all groups, our findings demonstrate an increase in the number of Hispanic women, women with a college degree, and women aged 30–39 years obtaining abortions. Our data also reflect the increase in the proportion of patients who were black. For all states, marital status remained stable across time. These trends are in line with other analyses conducted on women seeking abortions (Henshaw and Kost 2008; Jones et al. 2009).

Between 1994 and 2016, women from contiguous states at ≥ 20 weeks gestational age were an important subset of clients obtaining abortions in Georgia. While 2.0% of abortion procedures were at ≥ 20 weeks gestational age for Georgia residents, 13.8% of all contiguous state women obtained abortions at this gestational age. The higher proportion of abortions at ≥ 20 weeks gestational age for contiguous state residents may have contributed to the lower proportion of suction curettage for these patients (60.0%) compared to Georgia residents (83.7%) and a higher proportion of D&E procedures for contiguous state women (31.9%, compared to 9.8%). While other studies demonstrate that non-residents account for a large proportion of all abortions taking place at ≥ 20 weeks gestational age in Georgia (Roberts et al. 2015), our data points to a decline in the proportion of abortions at ≥ 20 weeks for contiguous state residents between 2012 and 2016. This decrease may reflect the impact of House Bill 954, the 2012 Georgia law banning abortions > 22 weeks gestational age (H.R. 954, 2012). For both populations, the increase in the proportion of mifepristone abortions is in line with general trends in medication abortion since its approval by the FDA in September 2000 (Gatter et al. 2015).

Contiguous state residents’ dependence on Georgia’s abortion-providing facilities, particularly for women ≥ 20 weeks gestational age, may stem from inequitable or insufficient services within their state of residency. Factors such as legal and insurance restrictions and provider availability often result in women traveling for abortion services (Barr-Walker et al. 2019). In 2011, Georgia had more clinics than any state in this review except for Florida and North Carolina. By 2017, Georgia’s 15 abortion-providing facilities surpassed North Carolina’s 14 facilities (Nash and Dreweke 2019). Yet women from both Georgia and contiguous states often lack close proximity to an abortion-providing facility. In 2014, median distances were 18.0 miles in Georgia, 26.9 miles in Tennessee, 26.2 miles in Alabama, 24.0 miles in South Carolina, 18.3 miles in North Carolina, and 7.8 miles in Florida (Bearak et al. 2017). Moreover, variations in scheduling, acceptability, and other factors may lead women to travel further than their nearest abortion-providing facility. These factors may have contributed to non-residents’ use of Georgia as a source of abortion services.

Understanding contiguous state women’s growing reliance on Georgia for abortion services alongside demographic, pregnancy, and abortion trends may help policymakers and providers better meet the needs of these women. Greater distances to facilities providing abortion services may lead to delays in seeking care (Jerman et al. 2017) and have important consequences post-procedure, such as a higher probability of visiting an emergency department and lower probability of seeking care at the point of service provision (Upadhyay et al. 2017). The additional costs associated with travel, which can include transportation, lodging, and childcare, also act as barriers to access abortion care (Barr-Walker et al. 2019). Abortion-related travel may have a greater impact on populations already experiencing disparities in health care access. Traveling further for services is especially common for women who are younger, have a lower socioeconomic status, are inhabitants of rural areas, or > 12 weeks gestational age (Barr-Walker et al. 2019). A better understanding of the demographics of women seeking abortion care may help practitioners better relay information about abortion care, link patients to abortion and post-abortion care services, and broaden unwanted pregnancy prevention efforts.

Finally, contiguous state women’s growing dependence on Georgia’s abortion-providing facilities has important implications within Georgia’s changing abortion policy environment. Between 1994 and 2015, Georgia had a gestational age limit of 26 weeks, which decreased to 20 weeks post fertilization in 2015 (Roberts et al. 2015). Between 2011–2017, Georgia also passed the fewest abortion restrictions compared to neighboring states (Ibid 2019). However, in May 2019, Georgia established “a detectable fetal heartbeat” as the gestational age limit for abortions (H.R. 481, 2019). While women travel for abortion services for myriad reasons, the elevated proportion of contiguous state women seeking abortions ≥ 20 weeks gestational age indicates that differing legally permissible gestational age limits may contribute to women’s decisions to seek care outside of their state of residency. Abortion restrictions in other states, such as Texas, have been associated with a decrease in abortions, overall reduction in abortion access (Grossman et al. 2014), increase in the distance to the nearest facility, and a higher probability of hardship related to obtaining services (Gerdts et al. 2016). However, more recent data claim that while abortion restrictions impact individuals’ access to services, they do not contribute to overall declines in abortions (Nash and Dreweke 2019). Given that the populations traveling for abortion services tend to be those who already experience disparities in health care access (Barr-Walker et al. 2019), the changing policy environment in Georgia may increase hardships for both residents and non-residents, resulting in additional health disparities. Moreover, while data on the chilling effects associated with changes to the legal status of abortion are lacking, confusion about legislation may also impact trends in women travelling for abortions.

This study had several limitations, including unknown values in the data set, the lack of data on county of residence and abortion event, and the inability to compare demographic characteristics between women receiving abortions and the general population. Another limitation includes the use of population data instead of individual data, which made it impossible to analyze the relationship between variables. However, this study was strengthened by the ability to analyze state-level surveillance data over a 23-year period.

## Conclusion

Our study demonstrates that contiguous state residents’ dependence on Georgia for abortion services increased during the period under review. While contiguous state and Georgia residents demonstrated similar trends in education, race, ethnicity, first pregnancy, and marital status, our findings suggest that contiguous state residents obtained abortions in Georgia at a later gestational age and received different abortion procedures compared to Georgia residents. Contiguous state residents sought more abortions in Georgia than delivery services, which may reflect a relative inadequacy of abortion services in their state of residency. Finally, contiguous state women’s growing dependence on Georgia for abortions indicates that policy changes affecting abortion availability in Georgia will likely affect women from other states.

It is still unclear how the recent changes in Georgia’s abortion laws may affect abortion-related travel to Georgia. Both qualitative and quantitative studies should evaluate how supportive or restrictive abortion policies may affect patients traveling for services. Future research should also include individual data to assess the relationship between demographic and abortion characteristics. This research may allow providers and policymakers to understand broader trends in abortion-related travel, including how changes in abortion availability in one state may affect both residents’ and non-residents’ access to services.
